# Three-dimensional biphase fabric estimation from 2D images by deep learning

**DOI:** 10.1038/s41598-024-59554-x

**Published:** 2024-04-18

**Authors:** Daniel Chou, Matias Etcheverry, Chloé Arson

**Affiliations:** 1 Georgia Institute of Technology, School of Civil and Environmental Engineering, Atlanta, GA 30332-0355 USA; 2 Ecole des Ponts Paris Tech, School of Applied Mathematics and Computer Sciences, 77420 Champs-sur-Marne, France

**Keywords:** Convolutional neural network, Microstructure analysis, 3D fabric descriptor, Stacked 2D images, Loss function, Civil engineering, Computational science, Composites

## Abstract

A pruned VGG19 model subjected to Axial Coronal Sagittal (ACS) convolutions and a custom VGG16 model are benchmarked to predict 3D fabric descriptors from a set of 2D images. The data used for training and testing are extracted from a set of 600 3D biphase microstructures created numerically. Fabric descriptors calculated from the 3D microstructures constitute the ground truth, while the input data are obtained by slicing the 3D microstructures in each direction of space at regular intervals. The computational cost to train the custom ACS-VGG19 model increases linearly with *p* (the number of images extracted in each direction of space), and increasing *p* does not improve the performance of the model - or only does so marginally. The best performing ACS-VGG19 model provides a MAPE of 2 to 5% for the means of aggregate size, aspect ratios and solidity, but cannot be used to estimate orientations. The custom VGG16 yields a MAPE of 2% or less for the means of aggregate size, distance to nearest neighbor, aspect ratios and solidity. The MAPE is less than 3% for the mean roundness, and in the range of 5-7% for the aggregate volume fraction and the mean diagonal components of the orientation matrix. Increasing *p* improves the performance of the custom VGG16 model, but becomes cost ineffective beyond 3 images per direction. For both models, the aggregate volume fraction is predicted with less accuracy than higher order descriptors, which is attributed to the bias given by the loss function towards highly-correlated descriptors. Both models perform better to predict means than standard deviations, which are noisy quantities. The custom VGG16 model performs better than the pruned version of the ACS-VGG19 model, likely because it contains 3 times (*p* = 1) to 28 times (*p* = 10) less parameters than the ACS-VGG19 model, allowing better and faster cnvergence, with less data. The custom VGG16 model predicts the second and third invariants of the orientation matrix with a MAPE of 2.8% and 8.9%, respectively, which suggests that the model can predict orientation descriptors regardless of the orientation of the input images.

## Introduction

Relating fabric tensors to the stiffness tensor is a long-standing issue in geomechanics^1^. A fabric tensor is, broadly speaking, a convolution of moments of probability density functions of microstructure descriptors. It can be a scalar, a vector, a matrix, or a tensor of higher order. Perhaps the most widely used fabric tensor in rock mechanics is the crack density tensor, initially defined by Oda^2^, who established a linear correlation between the first invariant of that fabric tensor and uniaxial compression strength in rock. In granular media, the principle of virtual work was invoked to relate the branch density tensor to the expression of the macroscopic stress tensor^3,4^. Joint invariants, defined as invariants of fabric tensors that are highly correlated to the stress invariants, were used to replace the stress invariants in the Dracker-Prager yield function, under the assumption of axial symmetry^5,6^. In those studies, the stress tensor was highly correlated to the fabric tensor that represents the statistical distribution of particle orientations. Zysset and Curnier^7^ derived an analytical expression of the elasticity tensor as a function of a general fabric tensor that represents the orientation distribution of directional dependent microstructure properties for isotropic, transverse isotropic and orthotropic materials. Later studies established correlations between the mechanical properties of salt rock and tensors that capture the magnitude and orientation of solidity, coordination, local solid volume fraction, and crack volume^8^.

X-ray Computed Tomography (XCT) scanning allows one to obtain stacks of 2D images that represent sections of a 3D material. XCT images are routinely used to reconstruct microstructures in 3D and to calculate statistical geometric features that can be used to define fabric tensors^9,10^. Kuo et al.^11^ calculated 2D fabric tensors from 2D binary images obtained in three orthogonal planes during XCT scanning and established a methodology to calculate 3D fabric tensors from those 2D fabric tensors. They assumed that the 3D fabric tensors were axially symmetric and that the principal values of the 3D fabric tensors were proportional to those of the 2D fabric tensors. To date, identifying 3D fabric descriptors from XCT images remains a challenge^10,11^.

Descriptors such as fabric tensors encapsulate a clear physical meaning, but they are chosen based on experience in a particular field of study^12,13^. Alternatively, the morphology and heterogeneity of microstructures can be quantified by means of correlation functions^14,15^. The N-point correlation function can accurately capture information of a dual phase microstructure. Deep learning has enabled huge advances in pattern detection, recognition and selection. It now has many applications, like in medical imaging. Resnet and the Visual Geometry Group (VGG) emerged as very powerful networks^16^, and transfer learning has shown a clear improvement in convergence time and result accuracy^17,18^. Although most problems treated with Resnet and the VGG pertain to image classification, it is easy to convert a model for regression and extract good results^19^. Thanks to the advancement of deep learning, 2-point correlation functions and descriptors have been used as input for microstructure reconstruction and can serve as proxy to measure the quality of a reconstruction in a generative model. To overcome the bias in the choice of descriptors, statistical methods were created to enable reconstruction with style data only^17,20,21^.

In the present study, we propose a deep learning approach to optimize the number of 2D slices in a 3D volume to achieve a targeted accuracy of 3D fabric tensor estimates in biphase media made of cemented aggregates. In “Data generation” section, we explain how we constructed virtual 3D microstructures, calculated associated 3D fabric descriptors that served as ground truth, and extracted 2D slice images that served as input data. In “Deep learning approach” section, we present two custom VGG models that take inputs of different formats, and we explain the protocol for training and testing. Our implementation is coded with python and Matlab^22^. We used Pytorch as our main machine learning module, and pytorch-lightning as a Pytorch framework. Our results are described and interpreted in “Results” section. The advantages, limitations and possible improvements to the models are discussed in “Discussion” section. Lessons learned and perspectives for future work are summarized in “Conclusions” section.

## Data generation

### Numerical construction of the virtual specimens

**Figure 1:**
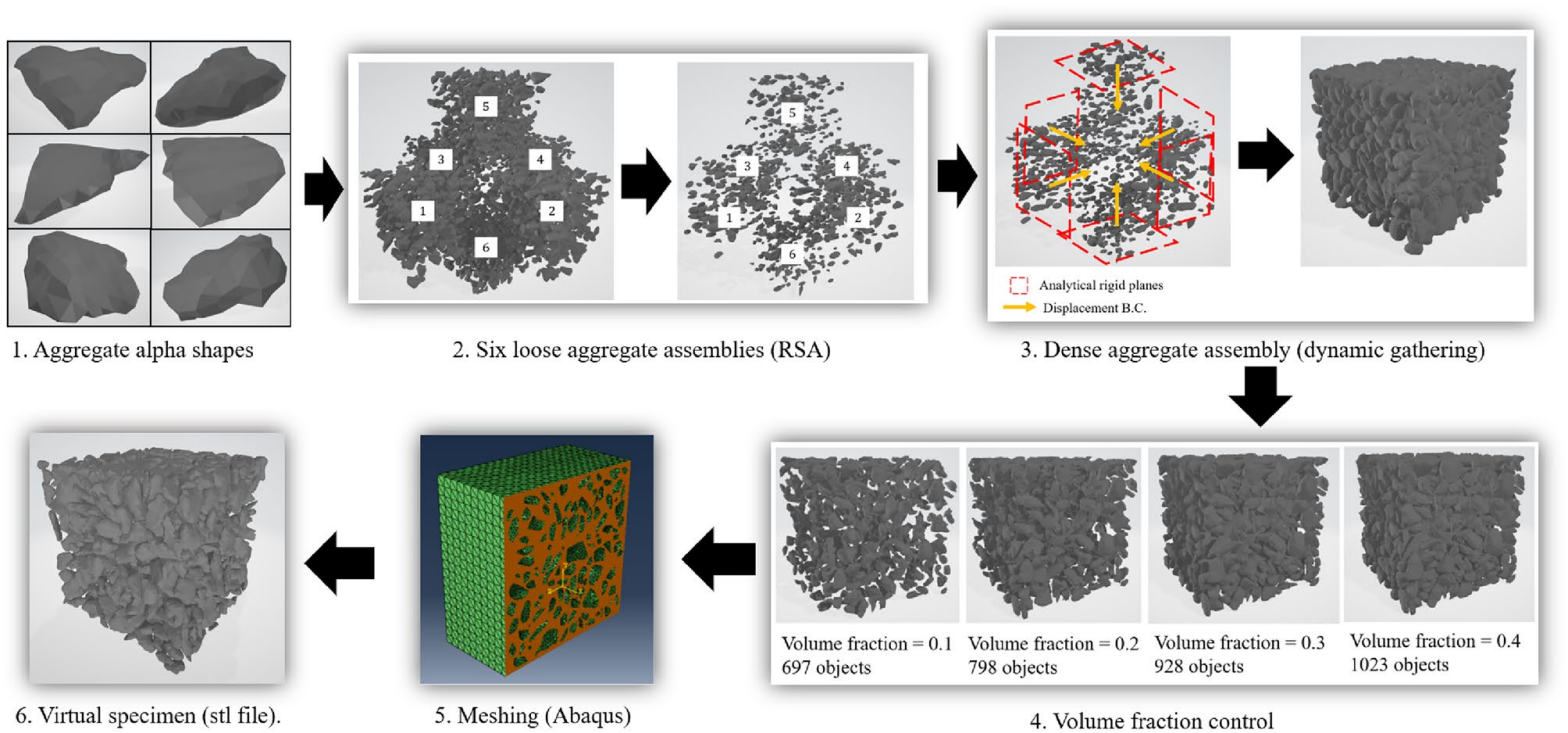
Method employed to generate three-dimensional biphase microstructures.

**Figure 2:**
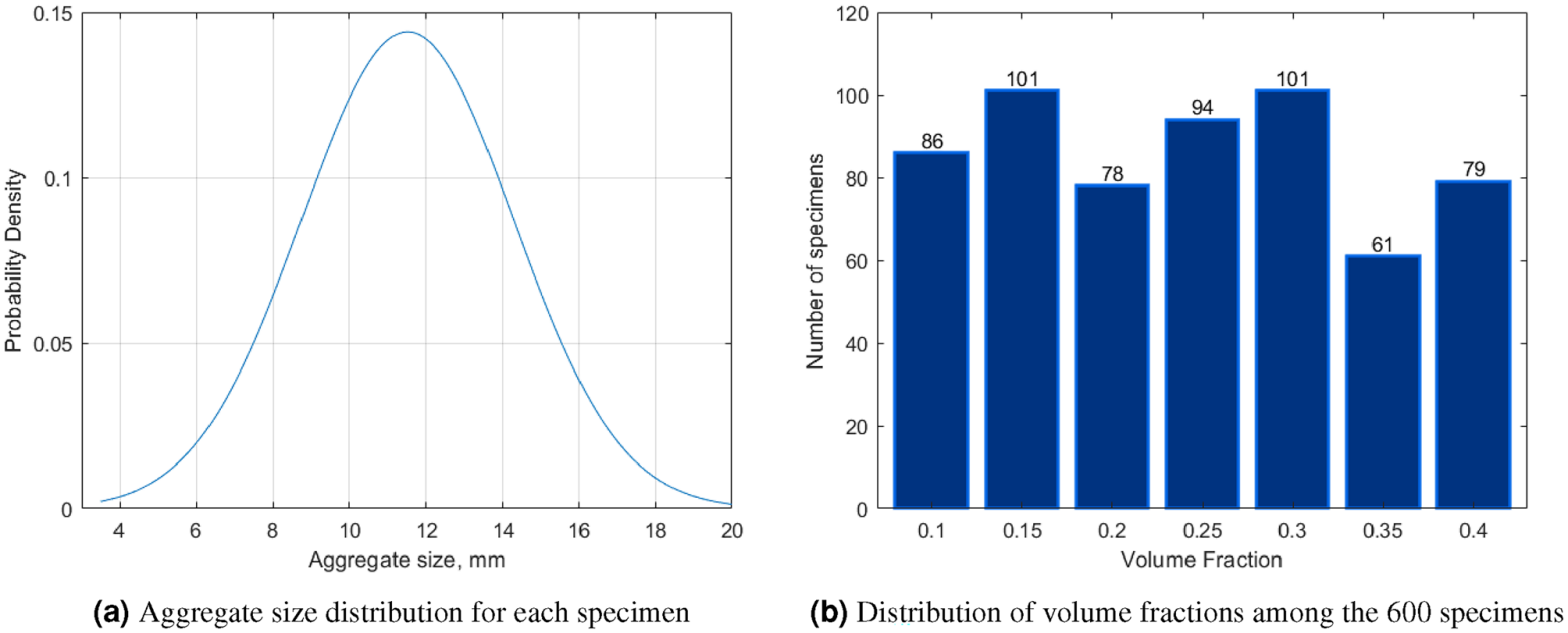
Controlled parameters of the numerically generated specimens.

Synthetic three-dimensional biphase microstructures were constructed to represent coarse aggregates embedded in a homogeneous matrix. The process to construct the numerical specimens is illustrated in Fig. 1. Aggregates were scanned and the resulting point clouds were transformed into solid alpha-shapes with Matlab (step 1). In total, 87 alpha-shapes were obtained from point clouds and stored in a database. Loose assemblies of aggregates were created with a Random Sequential Absorption (RSA) algorithm that sequentially and randomly picked alpha shapes from the database and fitted them in a cubic space (step 2). All shapes were equiprobable, and were scaled by the size distribution shown in Fig. 2a. The RSA algorithm takes the target volume fraction and a measure of exclusion distance as input. The RSA places objects randomly in a finite volume and rejects an object that is within the exclusion distance of another object previously fitted in that volume. The maximum volume fraction that can be reached iteratively with an RSA algorithm does not exceed 20%. Despite attempts to alter the exclusion distance criteria^23,24^ to improve the efficiency of the RSA algorithm, it remains difficult to generate high-density specimens. For that reason, we used the RSA algorithm to generate six cubes filled with loose aggregate assemblies, placed them along the sides of the target cubic domain, and used the Finite Element Method (FEM) to simulate the packing of the six loose assemblies into the target domain (step 3: dynamic gathering). These explicit dynamic simulations were conducted with Abaqus^25^. The loose assemblies were pushed by six rigid walls that were subjected to a controlled displacement (approximately 100 mm). The aggregates were modeled as rigid bodies with a mass density of $\rho$ = 2800 kg/m^3^, and a non-penetration condition was used at the contact between the aggregates. After dynamic gathering, the aggregate volume fraction was between 0.6 and 0.8. Target volume fractions of $V_f\in \{0.1,0.15,0.2,0.25,0.3,0.35,0.4\}$ were obtained by randomly removing aggregates from the aggregate assemblies obtained in step 3 (step 4). As a reference, the aggregate volume fraction in concrete used in construction is around 0.4. In total, 600 cubes filled with aggregate alpha-shapes were created, with the aggregate volume fractions shown in Fig. 2a. The 600 aggregate assemblies were imported into Abaqus, which was used to mesh the space between the aggregates, called matrix (step 5). Abaqus then automatically meshed the aggregates to match the mesh of the matrix. Lastly, the aggregate meshes were saved as .stl files, which can easily be opened in Matlab to calculate statistical microstructure descriptors (step 6).

### Generation of 2D images

**Figure 3:**
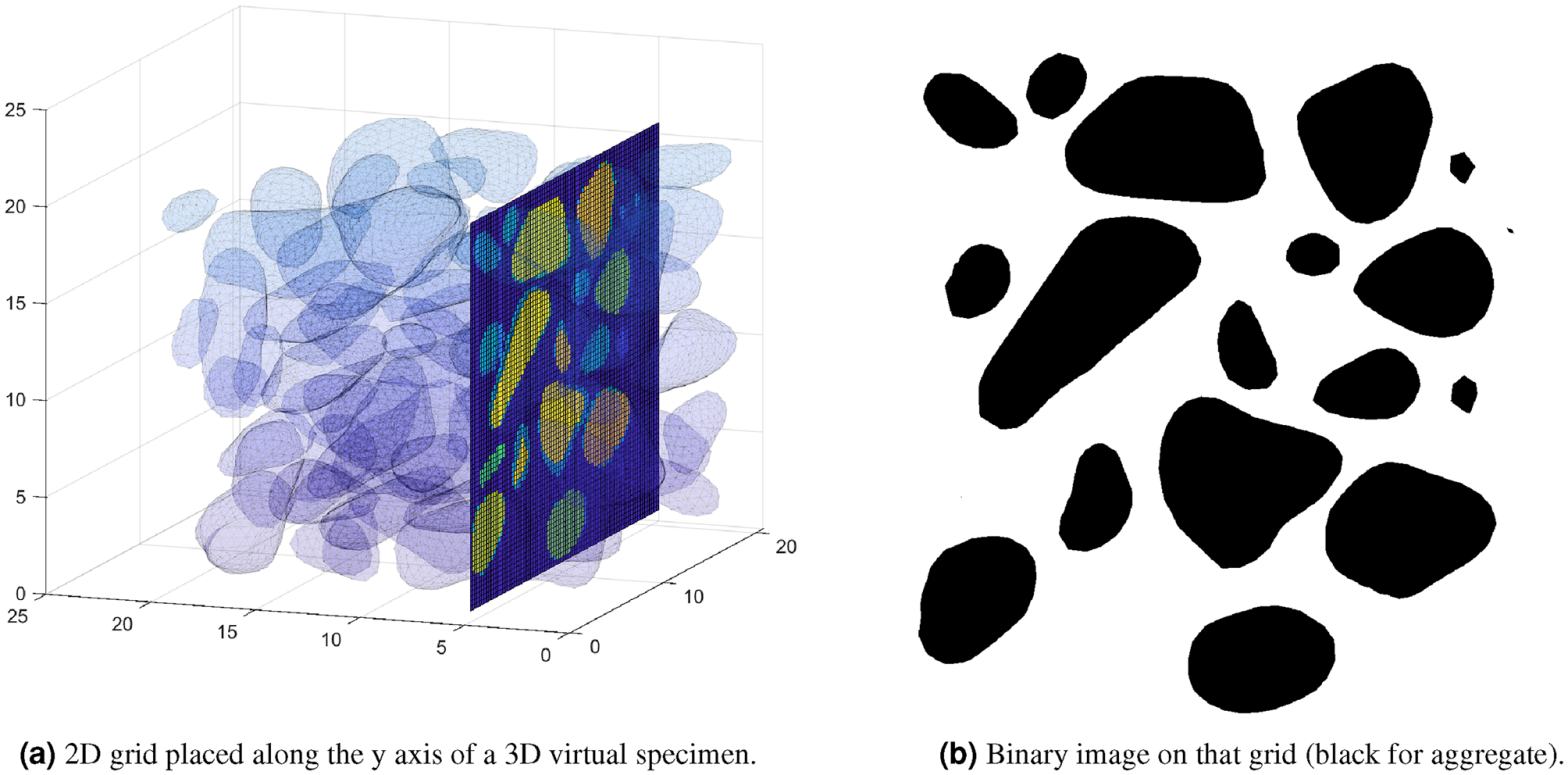
From a 3D microstructure to 2D grids and from 2D grids to binary images.

Regularly spaced two-dimensional images were extracted from the generated 3D microstructures in planes orthogonal to the x, y and z directions (Fig. 3). A 2D grid of squared elements was first created in the plane of interest (for example, at $y=y_0$ for the plane orthogonal to the y direction at position $y=y_0$). Each cell of the 2D grid was turned into a black pixel if the square was in an aggregate, and into a white pixel otherwise. The number of squares in the 2D grid thus equaled the number of pixels in the binary image. The position of the top left node of a square was the determining criterion to decide whether the square was in the aggregate phase or the matrix phase, since a square could lie at the interface between both phases. The Matlab module inpolyhedron^26^ was used to check whether the nodes of the 2D grid were located inside an aggregate or not. This algorithm has a very poor complexity: ${\mathcal {O}}(p \times N\times w \times h)$ with *p* the total number of images per direction, *N* the number of aggregates inside the virtual specimen, *w* and *h* the width and height of the 2D grid.

### Calculation of 3D fabric tensors (ground truth)

Here, the ground truth is a set of fabric tensors (scalars, vectors and second-order tensors) that describe the composition (e.g., aggregate volume fraction), dispersion (e.g., aggregate distance to nearest neighbor) and geometry (e.g., aggregate size, aspect ratios) of the features of the microstructure. By contrast with microstructure characterization approaches based on correlation functions or the Gaussian Random Field (GRF) method^27,28^, here, the fabric tensors are pre-defined and assigned a physical meaning, as explained below.

#### Principal component analysis (PCA)

By construction, each aggregate in the virtual specimen is a cloud of points (i.e., voxels). A principal component analysis (PCA) was performed on the vectors that connect each point of an aggregate to its barycenter. Each aggregate in the 3D microstructure is represented by a matrix $\underline{\underline{\mathbf {P^k}}}\in \mathbb {R}^{J_k\times D}$ that stores the positions of its points in reference to the barycenter. Here, *k* is an index that refers to the aggregate number in the specimen ($k\in \{1,2,3\ldots ,N\}$), $J_k$ is the number of points detected in the k^th^ aggregate, and *D* the space dimension (here, $D=3$). Noting $\underline{\underline{\textbf{C}}}_k\in \mathbb {R}^{D\times D}$ the covariance matrix of $\underline{\underline{\textbf{P}^\textbf{k}}}$, we obtain the eigenvalues ${{\varvec{\lambda }}}^{k}_{i}\in \mathbb {R}^{1\times 1}$ and eigenvectors $\underline{\textbf{v}}^{k}_{i}\in \mathbb {R}^{1\times D}$ of $\underline{\underline{\textbf{C}}}_k$ by solving the following equation:

1 $$\begin{aligned} \underline{\underline{\textbf{C}}}_k\cdot \underline{v}^{k}_{i}=\lambda ^{k}_{i}\underline{\textbf{v}^{k}_{i}} \end{aligned}$$

with $i\in \{1,\ldots ,D\}$. The eigenvector $\underline{\textbf{v}}^{k}_{i}$ associated with the greatest/smallest/intermediate eigenvalue $\lambda ^{k}_{i}$ defines the direction of the major/minor/intermediate axis of the k^th^ aggregate. The major/minor/intermediate semi-axis lengths are obtained by projecting the data points on the major/minor/intermediate axes, respectively, as follows:

2 $$\begin{aligned} \left\{ \begin{array}{c} a_k=\text {max}_j(\underline{\underline{\textbf{P}}}[j,:]\cdot \underline{\textbf{v}}^{k}_{1}) \\ b_k=\text {max}_j(\underline{\underline{\textbf{P}}}[j,:]\cdot \underline{\textbf{v}}^{k}_{2}) \\ c_k=\text {max}_j(\underline{\underline{\textbf{P}}}[j,:]\cdot \underline{\textbf{v}}^{k}_{3}) \\ \end{array} \right. \forall \, k \in \{1,2,3...,N\},\quad \forall \, j \in \{1,2,3...,J_k\} \end{aligned}$$

where $a_k$, $b_k$ and $c_k$ are respectively the semi -axis lengths of the major, intermediate and minor axes of the k^th^ aggregate, and where the eigenvalues are sorted in descending order: $\lambda ^{k}_{1}\ge \lambda ^{k}_{2}\ge \lambda ^{k}_{3}$.

#### Definition of the fabric descriptors

**Volume fraction**

One scalar descriptor denoted $v_f$ encodes the aggregate volume fraction (also called density in the following), calculated as the ratio between the volume of the aggregates by the volume of the cubic specimen.

**Size**

The size of an aggregate is defined as twice the length of the major semi-axis found by PCA. For the k^th^ aggregate: $G_k\,=\,2\,a_k$, where $a_k$ is defined in Eq. (2). At the scale of the specimen, we define two descriptors: the mean and standard deviation of the distribution $(G_k)_{k=1...N}$.

**Aspect Ratio**

We define two aspect ratios per aggregate: $b_k / a_k$ and $c_k / a_k$, where $a_k$, $b_k$ and $c_k$ are the lengths of the major, intermediate and minor semi-axes of aggregate k, found by PCA (see Eq. 2). At the scale of the 3D virtual specimen, we define four descriptors: the means and standard deviations of the distributions $(b_k/a_k)_{k=1...N}$ and $(c_k/a_k)_{k=1...N}$.

**Roundness**

The roundness descriptor encodes the elongation of the aggregates. The roundness $R_k$ of the k^th^ aggregate is defined as the ratio of the aggregate volume by the volume of its circumscribed sphere, of diameter $2\,a_k$ (see Eq. 2). At the scale of the specimen, we define two descriptors: the mean and standard deviation of the aggregate roundness $(R_k)_{k=1...N}$.

**Solidity**

The solidity $S_k$ of the k^th^ aggregate is defined as the ratio between the volume of the k^th^ aggregate and the volume of its convex hull. At the scale of the specimen, we define two descriptors: the mean and standard deviation of the aggregate solidity $(S_k)_{k=1...N}$.

**Orientation**

The unit eigenvector associated to the major eigenvalue of the k^th^ aggregate point cloud (calculated by PCA in “Principal component analysis (PCA)” section) is noted $\underline{\textbf{m}}_\textbf{k} = [m_{1, k},\ m_{2, k},\ m_{3, k}]$ in the global coordinate system ($\mathbf {e_1}$, $\mathbf {e_2}$, $\mathbf {e_3}$). The local orientation matrix of the k^th^ aggregate is defined as:

3 $$\begin{aligned} \begin{bmatrix} F_{k} \end{bmatrix} = \begin{bmatrix} m_{1, k}m_{1, k} &{} m_{1, k}m_{2, k} &{} m_{1, k}m_{3, k}\\ m_{2, k}m_{1, k} &{} m_{2, k}m_{2, k} &{} m_{2, k}m_{3, k}\\ m_{3, k}m_{1, k} &{} m_{3, k}m_{2, k} &{} m_{3, k}m_{3, k}\\ \end{bmatrix} \end{aligned}$$

The matrix $[F_{k}]$ is symmetric and can be encoded by six coefficients only: $F_{k, 11}, F_{k, 22}, F_{k, 33}, F_{k, 23}, F_{k, 13}, F_{k, 12}$. At the scale of the specimen, we define 12 descriptors: the means and standard deviations of each of the coefficients $F_{k, 11}, F_{k, 22}, F_{k, 33}, F_{k, 23}, F_{k, 13}, F_{k, 12}$ over the distribution of aggregates ($1 \le k \le N$). In order to encode frame-invariant information about the orientation of the aggregates, we also encode the second and third invariants ($I_2$ and $I_3$) of the average orientation matrix [*F*], as follows:

4 $$\begin{aligned} \begin{bmatrix} F \end{bmatrix} = \frac{1}{N}\sum _{k=1}^N [F_k] \end{aligned}$$

5 $$\begin{aligned} \left\{ \begin{array}{l} I_2 = (F_{11}F_{22} - F_{12}F_{12}) + (F_{22}F_{33} - F_{23}F_{23}) + (F_{11}F_{33} - F_{13}F_{13}) \\ I_3 = F_{11}F_{22}F_{33} + 2F_{12}F_{23}F_{13} - F_{22}F_{13}F_{13} - F_{11}F_{23}F_{23} - F_{33}F_{12}F_{12} \\ \end{array} \right. \end{aligned}$$

**Distance to nearest neighbor**

We compute the barycenter-to-barycenter distance between each aggregate k and its nearest neighbor, nd_k_. At the scale of the specimen, we define two descriptors: the mean and standard deviation of the distances to aggregate nearest neighbor (nd_k_)_k=1...N_.

#### Correlated descriptors

**Figure 4:**
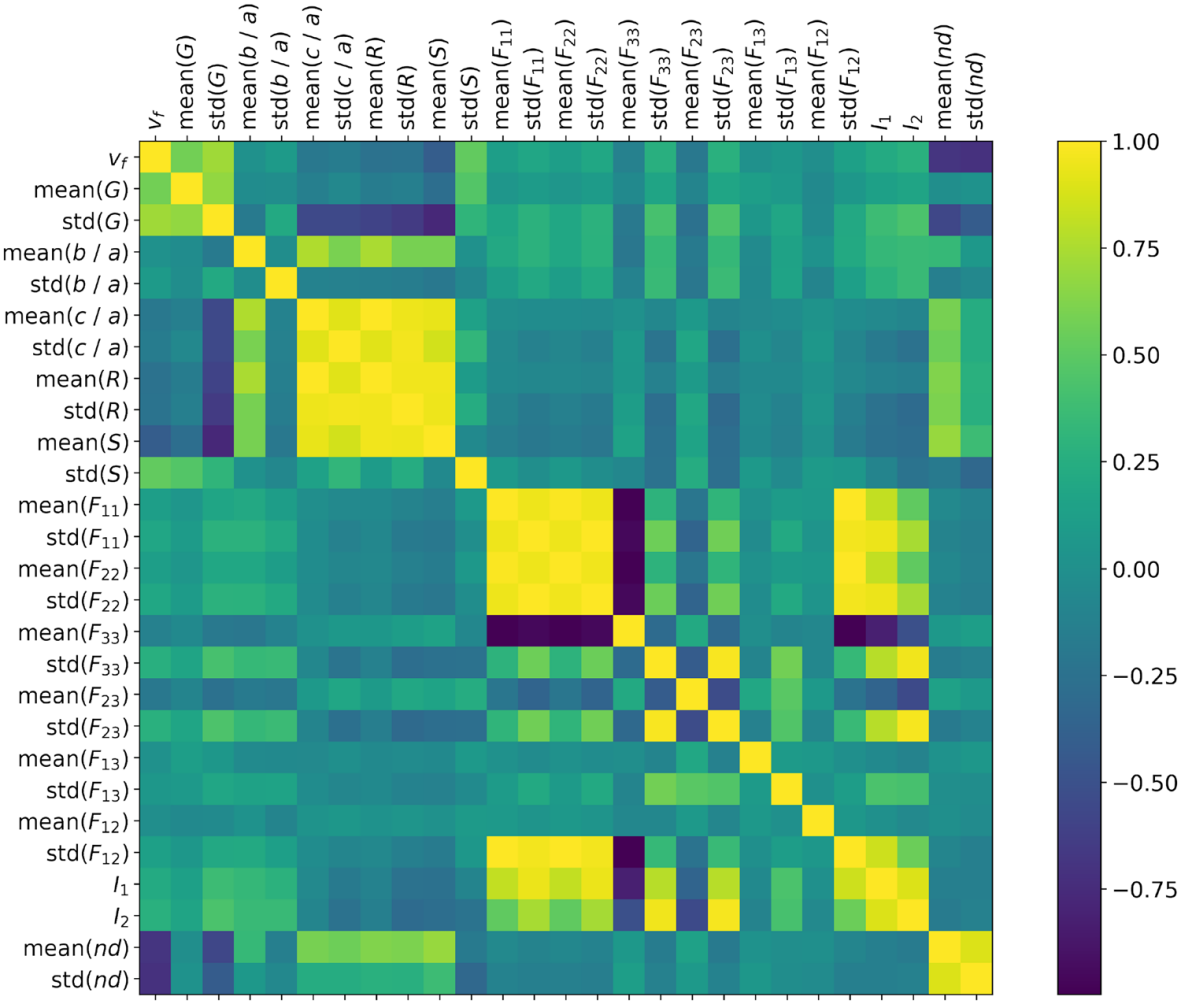
Correlation between descriptors on the whole dataset (600 virtual specimens).

Figure 4 shows the correlations between the 27 descriptors defined in “Principal component analysis (PCA)” section. The geometric aggregate descriptors (mainly size, aspect ratio, roundness and solidity) exhibit a high degree of correlation (close to 1). As expected, the aggregate volume fraction is negatively correlated to the average distance between an aggregate and its nearest neighbor. Of note, the non-diagonal coefficients of the average aggregate orientation tensors ($F_{12}$, $F_{23}$ and $F_{31}$) are not correlated to any other descriptor, likely because the non-diagonal terms of the aggregate orientation tensors are close to zero. The values of $F_{12}$, $F_{23}$ and $F_{31}$ exhibit a low magnitude and a low variance, which suggests that the non-diagonal terms of the orientation tensors will be difficult to estimate with a deep learning algorithm. We will test this hypothesis in the performance assessment presented in “Results” section.

## Deep learning approach

We developed two deep learning strategies to estimate all or part of the 27 fabric descriptors defined in “Principal component analysis (PCA)” section from sets of 2D images extracted from the 3D microstructure in orthogonal planes. We used Convolutionnal Neural Networks (CNN) because they are particularly suitable for image datasets, and we worked with the VGG, because VGG networks are pre-trained to find interesting patterns in $224\times 224$ RGB images. Pre-training allows better and faster convergence by transfer learning. The VGG appeared in 2014 and was used in classification tasks, notably in the ImageNet Large-Scale Visual Recognition Challenge, where it beat state-of-the-art models like GoogleNet^29^. VGG models have also been used extensively for image style transfer and 2D micro-structure analysis^30–32^. The first algorithm that we tested takes three 3D images (i.e., three stacks of 2D images) as input, whereas the second algorithm takes three channels of 2D images (i.e., three concatenated 2D images) as input. We assessed the performance of the deep neural networks when 1, 3, 5 or 10 images are extracted along each spatial direction. In the following, we note *p* the number of 2D images per direction.

### Model 1: three stacks of 2D images as input

#### Structure of the CNN

Model 1 is based on the pretrained VGG19 network^29–31^ and it is designed to calculate the 27 fabric descriptors defined in “Principal component analysis (PCA)” section, except: the invariants $I_2$ and $I_3$; the mean and standard deviation of the distribution of distances to nearest neighbor. In total, Model 1 was thus trained to estimate 23 descriptors, which were concatenated into a vector of dimensions ($1 \times 23$). The original VGG19 model is composed of 16 convolutional layers and 3 fully connected (FC) layers. We only kept the convolutional layers before the third max pooling layer, which are critical for 2D microstructure image characterization^31^. The convolutional layers of the original VGG19 model comprise 20,024,384 trainable parameters, while the convolutional layers of the pruned VGG19 model comprise only 1,145,408 trainable parameters, which reduces the training time significantly and allows running the calculations on the open access platform Kaggle (see Table 1).

**Table 1:** Computational constraints of Kaggle platform (as of Fall 2022).

Session launch time	Storage disk space	CPU RAM	GPU memory	GPU quota
12 h at once	73 GB	13 GB	15.9 GB	30 h/week

CNNs take 2D images as input. Axial-Coronal-Sagittal (ACS) convolutions are applied to the convolutional layers of the pruned VGG19 model in order to use 3D images as input. We used the ACSConv package, which was initially developed to handle 3D medical data sets. The ACS conversion makes it possible for a 2D CNN model to process a 3D data set without increasing the number of trainable parameters of the convolutional layers^33^. Changing the number of images (*p*) extracted in each direction of the 3D microstructure only affects the number of parameters of the FC layers (see Table 2).

**Table 2:** Number of trainable parameters in the pruned ACS-VGG19 model, as a function of the number of images *p* extracted in each direction of the 3D microstructure.

p	CNN	FC	The whole model
1	1,145,408	51,416,343	52,561,751
3	1,145,408	154,176,791	155,322,199
5	1,145,408	256,937,239	258,082,647
10	1,145,408	513,838,359	514,983,767

**Figure 5:**
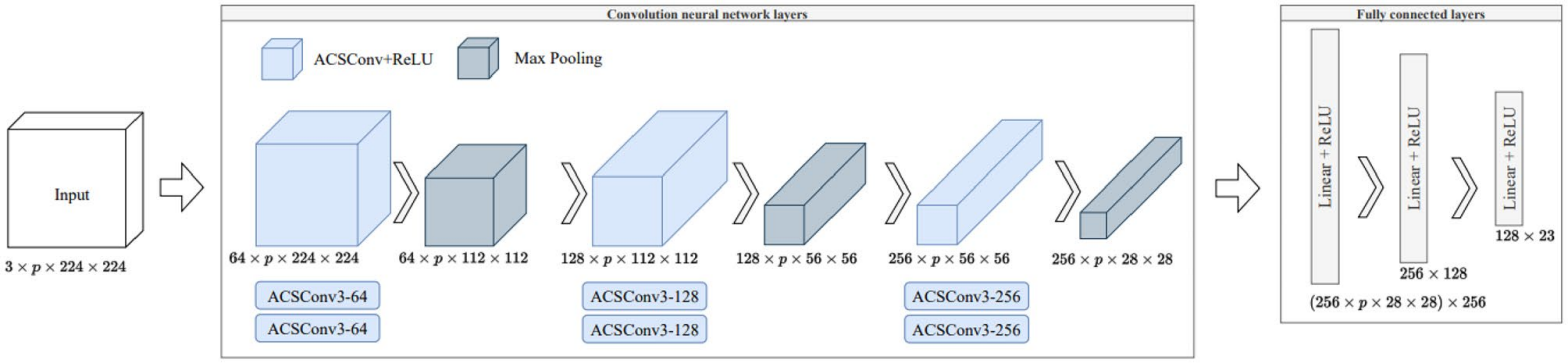
The structure of the pruned ACS-VGG19 model (Model 1).

The structure of the pruned ACS-VGG19 model adopted here is illustrated in Fig. 5. The network is made of 20 layers, including:

- Fifteen convolutional layers, organized in three convolutional blocks that each contain a first convolutional layer followed by a ReLU layer, a second convolutional layer followed by a ReLU layer, and a max pooling layer;
- A FC block that contains three linear layers separated by ReLU activation layers (two ReLU activation layers total).

#### Format of the input data

**Figure 6:**
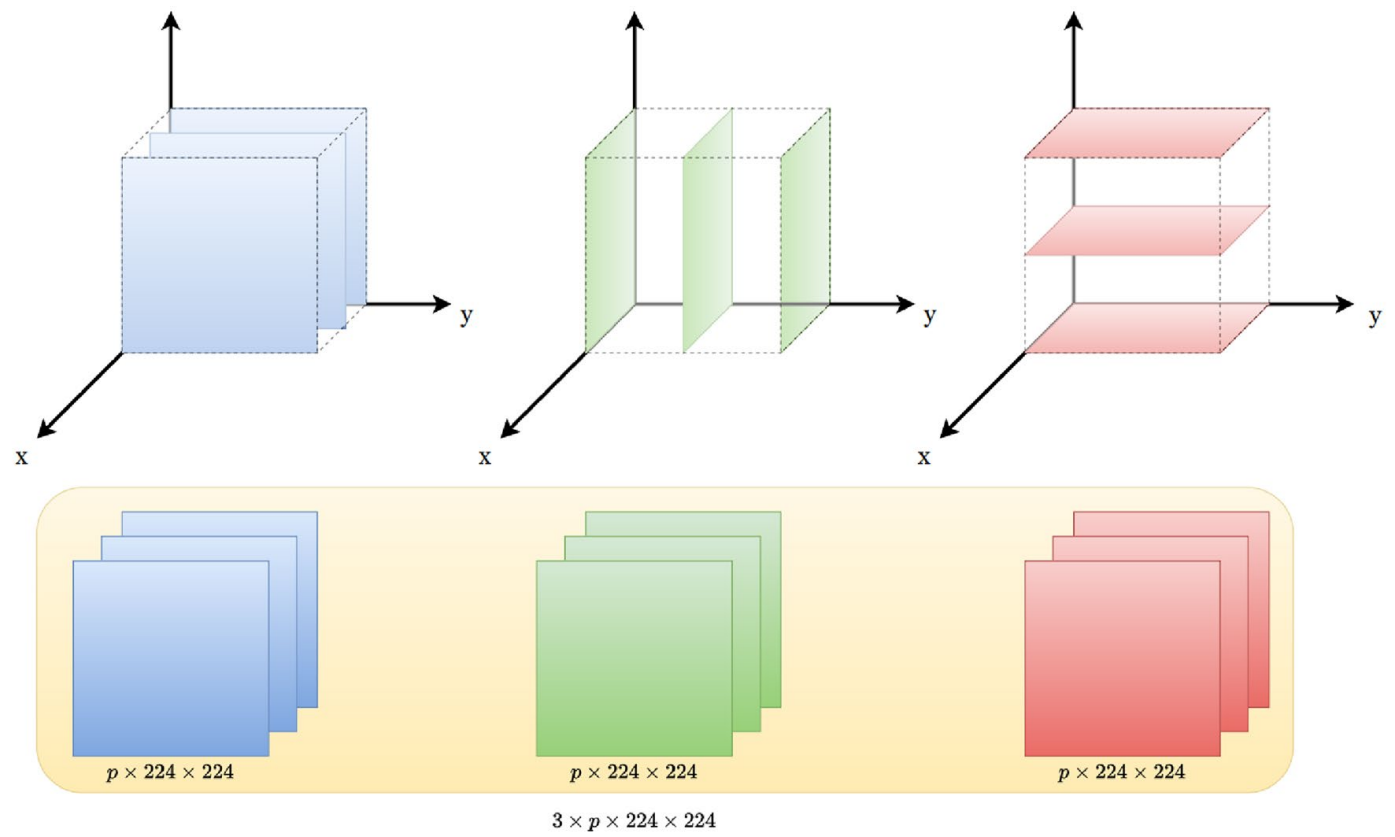
Format of the input data for Model 1: concatenated *p*-stacked arrangement of 2D images.

Figure 6 illustrates how 2D images extracted in planes orthogonal to the x, y and z directions are assembled into a triplet of stacks of depth *p*, and how these triplets are then concatenated to form a unique input tensor of dimensions ($3 \times p \times w \times h$) as input. The input tensor stores *p* stacked images in 3 orthogonal directions of space, and each image has a width *w* of 224 pixels and a height *h* of 224 pixels.

#### Data splitting

Each of the 600 virtual specimens provides two data sets: 23 ground truth fabric descriptors and 2D images extracted in three orthogonal directions of space. We use 60% of the specimen data for training, 20% for validation and 20% for testing. The training data set is used to update the parameters of the model to minimize the training loss at each iteration. The validation data set is used during training to calculate the loss for estimating unseen data and adjust hyperparameters so as to optimize the learning curve. The testing data set is used after training to assess the performance of the model in predicting unseen data. The assessment is based on a comparison between the fabric descriptors estimated during testing and the ground truth descriptors of the testing data set.

#### Pre-processing, measure of error and hyperparameters

The loss functions measure the distance between estimated and ground-truth fabric descriptors. In order to use the same weight for each fabric descriptor, we applied a minimum-maximum normalization to each fabric descriptor. For a descriptor *X*, the normalized descriptor $\overline{X}$ is $(X-X_{min})/(X_{max}-X_{min})$.

We assessed the performance of Model 1 with three different loss functions: the Mean Square Error (MSE), the Root-Mean-Square Error (RMSE) and the Mean Absolute Error (MAE) , which are defined as follows:

6 $$\begin{aligned} \text {MSE}\,&=\,\frac{1}{N_{tot}}\sum _{nd}(y_{nd}-\hat{y}_{nd})^2 \end{aligned}$$

7 $$\begin{aligned} \text {RMSE}\,&=\,\sqrt{\text {MSE}} \end{aligned}$$

8 $$\begin{aligned} \text {MAE}\,&=\,\frac{1}{N_{tot}}\sum _{nd}|y_{nd}-\hat{y}_{nd}| \end{aligned}$$

in which $y_{nd}$ and $\hat{y}_{nd}$ are the ground-truth and estimated values of the d^th^ fabric descriptor (one of the 23 fabric descriptors under study) in the n^th^ estimation.

A stochastic gradient descent (SGD) algorithm is employed. While the gradient descent algorithm updates model parameters after calculating the loss for the whole training set, the SGD updates the model parameters based on the loss of a single data point, picked randomly in the training set. The stochasticity of the SGD algorithm accelerates convergence and avoids overfitting. The main equations of the SGD algorithm are:

9 $$\begin{aligned}&\underline{\underline{{\textbf{w}}}}^{k+1}=\underline{\underline{{\textbf{w}}}}^k-\eta (\nabla L^k-2\lambda ||\underline{\underline{{\textbf{w}}}}^k||^2_2) \end{aligned}$$

10 $$\begin{aligned}&\left\{ \begin{array}{cc} \underline{\underline{{\textbf{v}}}}^k=\gamma \underline{\underline{{\textbf{v}}}}^{k-1}+\eta \nabla L^k \\ \underline{\underline{{\textbf{w}}}}^{k+1}=\underline{\underline{{\textbf{w}}}}^k -\underline{\underline{{\textbf{v}}}}^k \end{array} \right. \end{aligned}$$

The matrix $\underline{\underline{{\textbf{w}}}}$ is the weight matrix to be updated, and $\underline{\underline{{\textbf{v}}}}$ is coined as the velocity. The learning rate $\eta$ controls the step size. The weight decay $\lambda$ is used to avoid overfitting. The momentum $\gamma$ avoids locking the solution in a local optimum. The values of the hyperparameters are adjusted by trial and error to improve performance. Table 3 summarizes the values of the hyperparameters used in this study.

**Table 3:** Hyper-parameters used in Model 1.

Batch size	Termination error	Learning rate, $\eta$	Momentum, $\gamma$	Weight decay, $\lambda$
8	0.001	–	0.9	0.0005

Since the convolutional layers of the VGG19 model are pre-trained, we compared two strategies: (i) Only the parameters of the FC layers of Model 1 are trained with the input data set of this study. The convolutional layers are fixed, i.e., we are fixing all the parameters of the convolutional layers to the values obtained during pre-training. (ii) The parameters of all the layers of Model 1 are trained with the input data set of this study. The convolutional layers are trainable, i.e., the parameters learned during pre-training are recalculated during training.

### Model 2: Three channels of concatenated 2D images as input

#### Structure of the CNN

Model 2 is based on the pretrained VGG16 network^34^ and it is designed to estimate all 27 fabric descriptors defined in “Principal component analysis (PCA)” section. VGG16 is composed of five convolutionnal blocks and of three fully connected layers:

- The first two convolutional blocks are each composed of two 2D convolutional layers each followed by a ReLU activation layer, and a 2D max pooling layer that divides the width and height of the output by 2.
- The following three convolutional blocks are each composed of three 2D convolutional layers each followed by a ReLU activation layer, and a 2D max pooling layer.
- The two first fully connected layers have 512 neurons.
- The last fully connected layer has 27 neurons.

**Figure 7:**
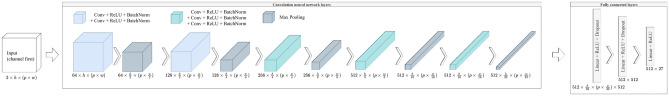
The structure of the custom VGG16 model (Model 2).

VGG16 was originally used for classification tasks. Its output is a vector of length 1,000, each entry representing the probability of the input image to belong to a certain class. In our study, the goal is to conduct a regression to predict 27 continuous values. Thus, we replaced the original fully connected layers in VGG16 by three fully connected layers: the first two layers have 512 neurons while the third layer has 27 neurons. No further activation was applied. Two dropout layers were applied between the 3 fully connected layers, to avoid overfitting. The structure of the VGG model adopted here is illustrated in Fig. 7. The convolutional layers of VGG16 use filters with a very small receptive field: a $3\times 3$ kernel with a stride of 1 and a padding of 1. The number of filters increases up to 512. We applied a batch normalization layer^35^ between each 2D convolutional layer and its activation function to accelerate the training process.

#### Format of the input data

**Figure 8:**
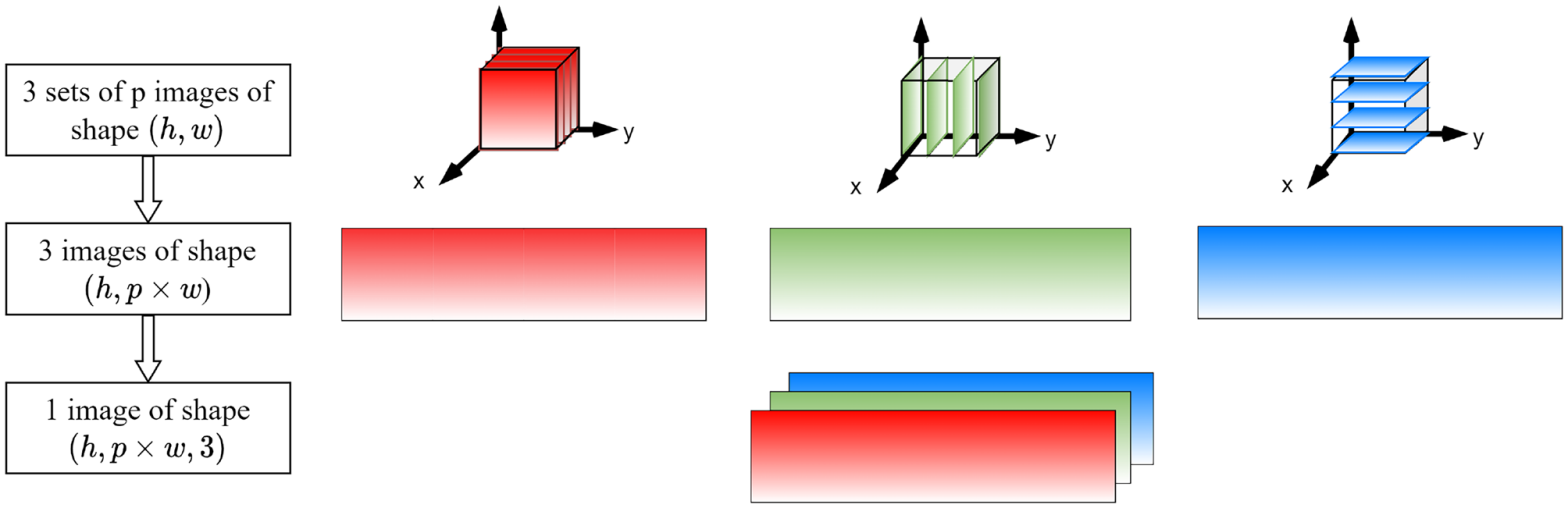
Format of the input data for Model 2: stack of *p*-concatenated arrangement of 2D images.

**Figure 9:**
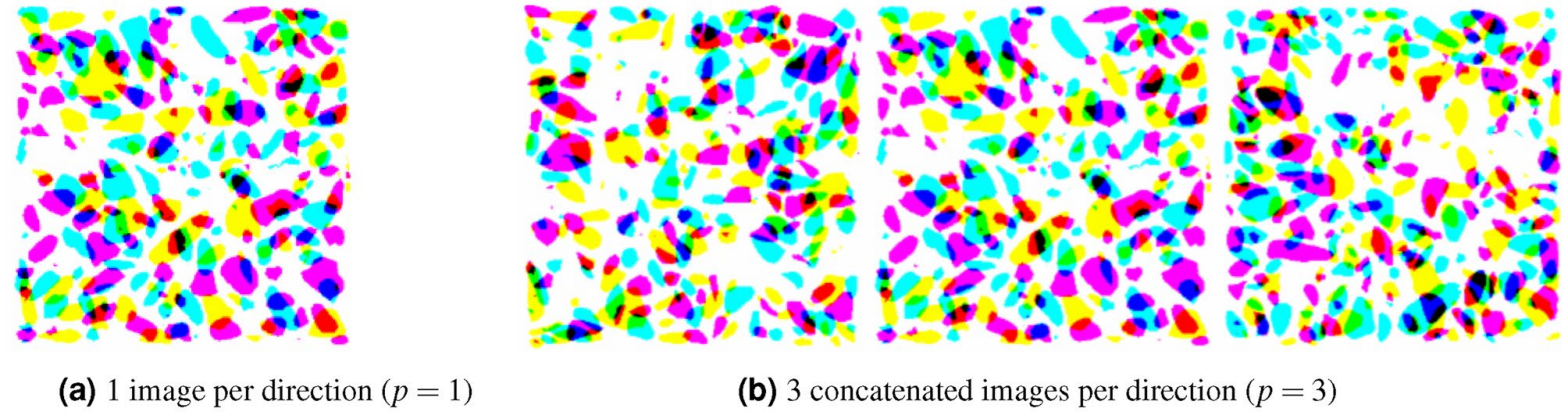
Examples of *p*-concatenated arrangements stacked in 3 channels.

The input 2D images are arranged in stacks of *p*-concatenated images, as shown in Fig. 8. All the images extracted along the same axis are concatenated along the width, resulting in three images of shape $(h, p\times w)$, which represent three channels of input data. The three channels are stacked along a third dimension, the depth, such that the input image has dimensions $(h, p\times w, 3)$. Each channel represents multiple images taken along the same axis and have a similar function as a RGB color channel. The *p*-concatenated arrangement has the advantage of being interpretable by any convolutional network. Figure 9 shows examples of concatenated arrangements stacked in 3 channels for $p=1$ and $p=3$.

#### Data splitting

Here, we adopt a 70-30$\%$ data splitting, which we obtained with the scikit-learn function train_test_split. The 27 fabric descriptors defined in “Principal component analysis (PCA)” section are calculated separately for the 420 microstructures that are in the training dataset and for 180 microstructures that are in the testing dataset. We checked that the test and train distributions were statistically equal by using a Kolmogorov-Smirnov test.

#### Pre-processing, measure of error and hyper-parameters

Before training, we instantiated a pre-trained VGG16 model with 3-concatenated images as inputs. The cross-sectional binary images extracted from the 3D microstructures have dimensions (64, 64), so the input images had dimensions (64, 192, 3). Although the original input size of VGG16 is (224, 224, 3), we can still apply it to images of size (64, 192, 3), by using smaller-sized fully connected layers. In each of the three stacked channels, the mean pixel value is subtracted from the image, and a Gaussian blur is applied before training the model. The 27 target descriptors are all normalized so that they all fit in [0, 1]^36^.

The Mean Absolute Error (MAE) is used as a loss. We compute the MAE on every batch of the training data, to minimize it on every batch of pair (image, descriptors). The Mean Absolute Percentage Error (MAPE) is used as a complementary performance metric. We compute the MAPE on the whole test data. The MAE and MAPE are defined as follows:

11 $$\begin{aligned} \text {MAE} = \frac{1}{N_{tot}} \sum _{i,d} |y_{i, d} - \hat{y}_{i, d} |,\qquad \text {MAPE} = \frac{100}{N_{tot}}\sum _{i,d} \left| \frac{y_{i, d} - \hat{y}_{i, d}}{y_{i, d}} \right| \end{aligned}$$

in which $y_{i, d}$ and $\hat{y}_{i, d}$ are the *d*-th target and predicted descriptor of the *i*-th microstructure, and $N_{tot}$ is the size of the test dataset. We implemented a naive model, coined as mean algorithm, which simply computes the mean descriptors in the training dataset and outputs those mean descriptors for every microstructure in the test dataset. The predictions of the mean algorithm provide a baseline to which the performance of Model 2 can be compared.

We used Adam optimizer with a learning rate of $1\times 10^{-4}$, trained for 700 epochs, with 64 samples per batch. The model was trained using the NVidia K80 GPU provided by Kaggle.

## Results

### Model 1: three stacks of 2D images as input

#### Performance based on computational cost

The number of trainable parameters in the convolutional layers is one to two orders of magnitude smaller than the number of trainable parameters in the fully connected layers (Table 2). One could wonder whether fixing the convolutional layer parameters to their pre-training values impacts the training time in the same order of magnitude. Table 4 shows that fixing the convolutional layers divides the required training time by two, regardless of the number of input images. This observation indicates that in comparison to the FC layer parameters, the convolutional layer parameters are more expensive to train. In the following, we evaluate whether there is a performance cost associated with the computational savings, by comparing the loss of Model 1 with trainable and fixed convolutional layers.

**Table 4:** Training time for ACS-VGG layers at termination epoch = 75, unit: seconds.

Loss function	$p=1$	$p=3$	$p=5$	$p=10$
Trainable CNN				
MSE	776.21	2346.01	3947.4	7843.29
RMSE	783.01	2348.79	3937.54	7850.41
MAE	786.26	2345.01	3949.63	7843.91
Fixed CNN				
MSE	371.25	1122.96	1920.7	3754.45
RMSE	371.36	1127.22	1891.42	3757.84
MAE	370.76	1124.33	1890.63	3761.43

#### Performance based on loss (error)

The values of the loss functions for all the training settings are summarized in Table 5. First, we note that the variation of the loss with *p* is not monotonic in any of the training settings. Fixing the convolutional layers does not significantly lower the loss: in most settings, the loss increases by 5% to 12% when the convolutional layers are fixed, except for the MSE with $p=10$, the MAE with $p=3$ and the MAE with $p=10$. From Table 4, training the model with fixed convolutional layers takes half the time required for training the fully trainable model. The trade-off is an increase in loss that, in most configurations, does not exceed 7%. We conclude that the training setting with fixed convolutional layers is the most advantageous for the purpose of this study. For fixed convolutional layers, the best performance is achieved for $p=10$. For example, the MAE decreases by 6% when changing the input from $p=1$ to $p=10$ with fixed convolutional layers. However, the MSE and RMSE do not decrease monotonically as *p* increases. Since the computational time roughly increases linearly with *p* (see Table 4), we conclude that the best trade-off between minimization of loss and minimization of computational time is when the model is trained with fixed (pre-trained) convolutional layers, and one image taken in each plane ($p=1$).

**Table 5:** Model 1 loss values for the testing data set.

Loss function	$p=1$	$p=3$	$p=5$	$p=10$
Trainable CNN				
MSE	0.0415	0.0411	0.0392	0.0408
RMSE	0.1928	0.1916	0.1906	0.1912
MAE	0.1422	0.1453	0.1397	0.1437
Fixed CNN				
MSE	0.0426	0.0465	0.0429	0.0393
RMSE	0.1978	0.1941	0.1955	0.1915
MAE	0.1489	0.1413	0.1414	0.1397

**Figure 10:**
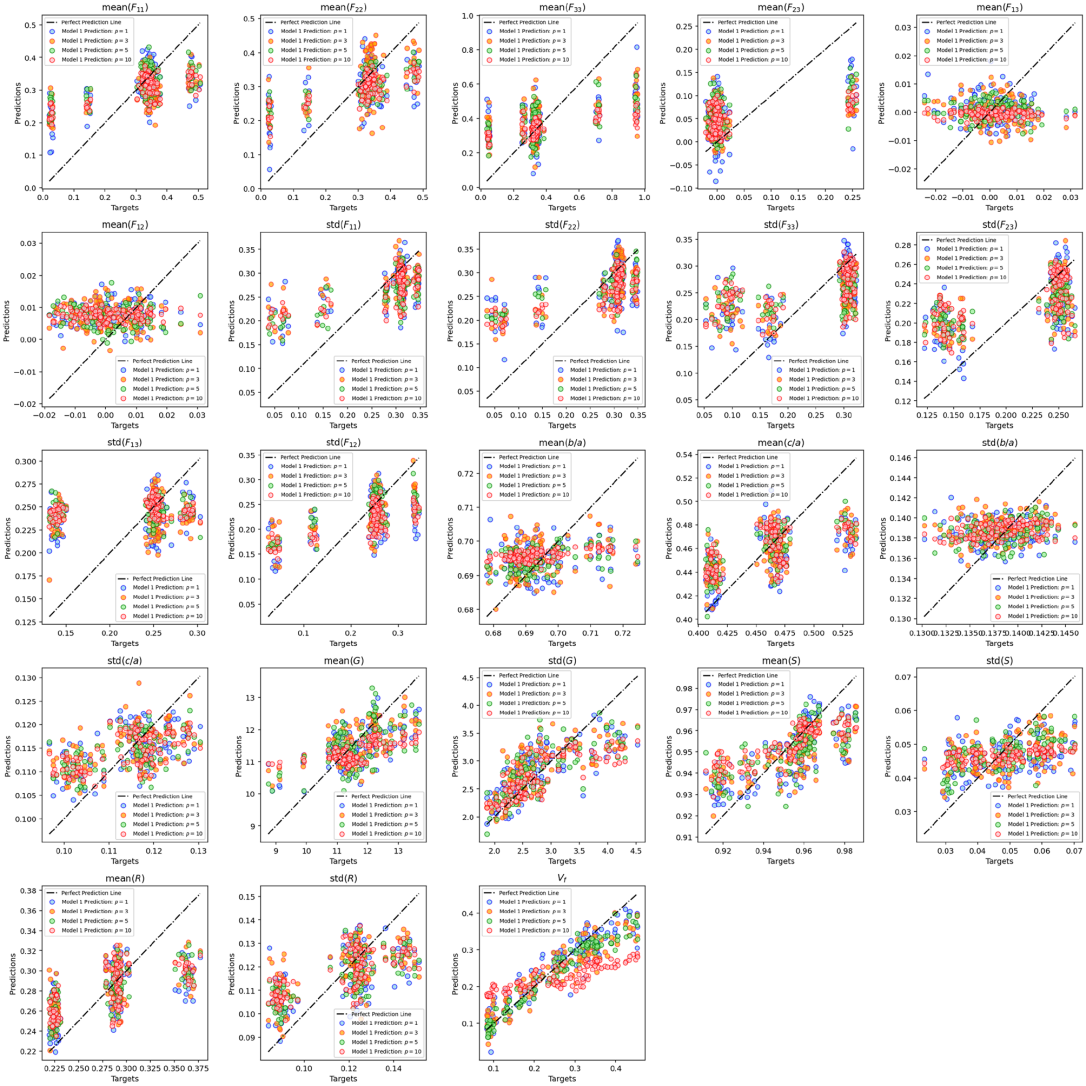
Microstructure descriptors predicted with Model 1 during testing versus ground truth.

**Figure 11:**
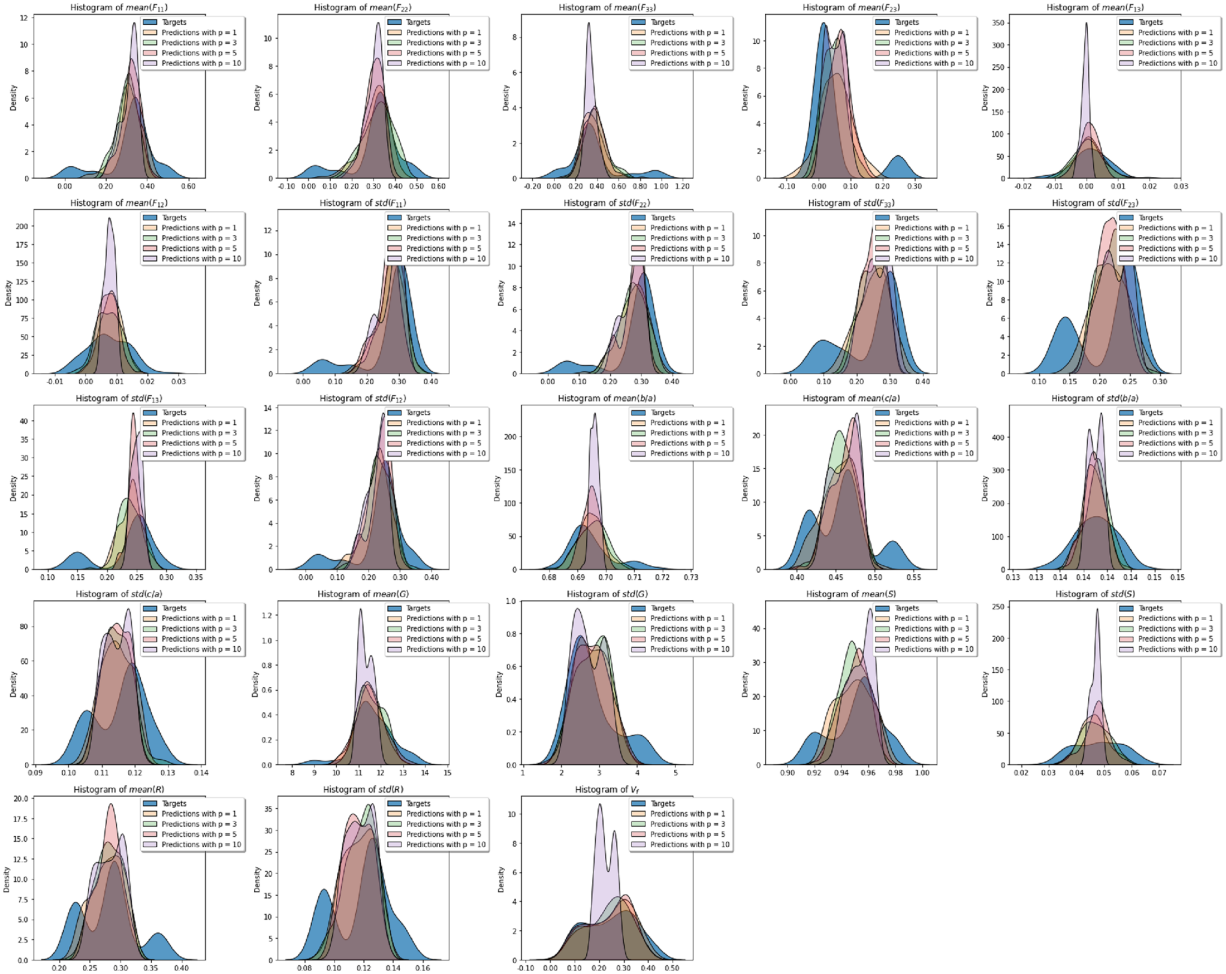
Distributions of the target and predicted descriptors in the test dataset after training Model 1 with different p values. Microstructure descriptors are not normalized in these plots.

The value of each microstructure descriptor predicted with Model 1 during testing is plotted against its ground truth value for all the number of slices considered in Fig. 10. Figure 11 shows the distributions of the target and predicted descriptors for trainable convolutional layers and different *p* values. The features have been converted to their actual values (instead of their normalized values) for a better physical understanding. The overlap between the target and predicted descriptors varies largely across descriptors. To better assess the large predictability discrepancy between features, we define a new metric of accuracy in “Performance based on accuracy” section.

#### Performance based on accuracy

**Figure 12:**
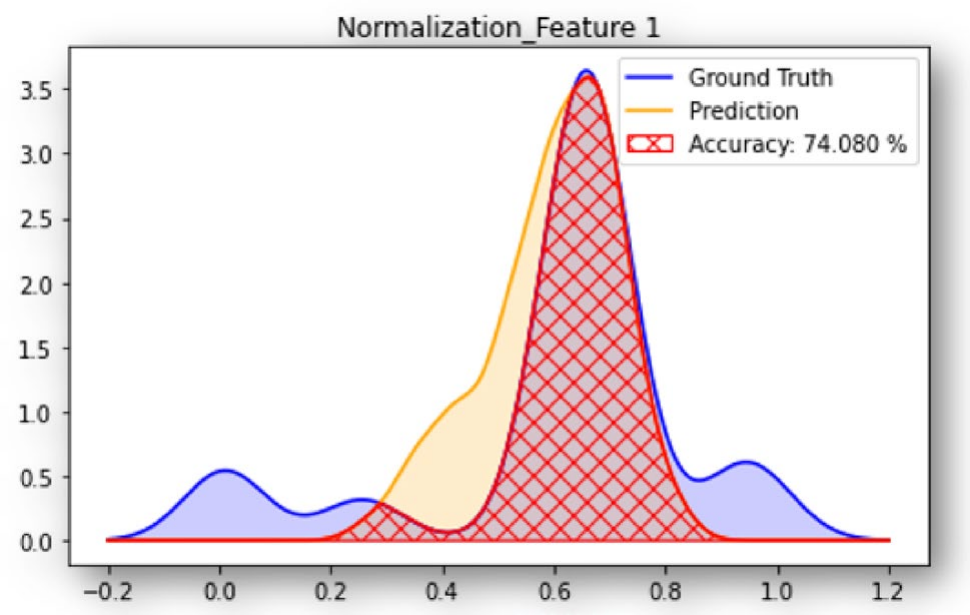
Accuracy of Model 1 for estimating $F_{11}$ from the training data set (MSE loss function, fixed convolutional layers, $p=1$).

Similar to the MAPE used as complementary performance metric in Model 2, we define a complementary measure of accuracy in Model 1. The accuracy of Model 1 for the i^th^ fabric descriptor is defined as the ratio between the area intersected by the ground-truth and estimated distribution curves of the i^th^ feature and the area under the ground-truth distribution curve of that feature, as illustrated in Fig. 12.

The accuracy of Model 1 averaged over all 23 features is presented in Fig. 6 for all the configurations tested: 1, 3, 5 or 10 images per axis; MSE, RMSE or MAE loss function; trainable or fixed convolutional layers. The MAE is the loss that gives the most consistent predictions in that setting, since it decreases as *p* increases. However, overall, increasing the number of images per axis (*p*) increases the computational cost (Table 4) but does not improve the mean accuracy of the model significantly (Table 6). The highest mean accuracy that the model can reach during testing is $68.65\%$. It is obtained with the MSE loss function and for 1 image per axis. This somewhat surprising result may be attributed to an insufficient training data set and/or to the complexity of the prediction task, which consists in estimating 23 features simultaneously, and to the low values of the second-order moments of probability (i.e., standard deviations) of the fabric descriptors, which are difficult to predict because they are similar to noise.

**Table 6:** Average accuracy of Model 1 ($a\%$).

Loss function	$p=1$	$p=3$	$p=5$	$p=10$
Trainable CNN				
MSE	68.65	66.19	62.90	52.78
RMSE	65.01	56.30	50.42	52.22
MAE	64.76	54.88	54.43	50.64
Fixed CNN				
MSE	67.24	67.82	67.25	63.18
RMSE	64.24	57.76	54.89	55.39
MAE	63.46	60.38	55.27	56.90

**Figure 13:**
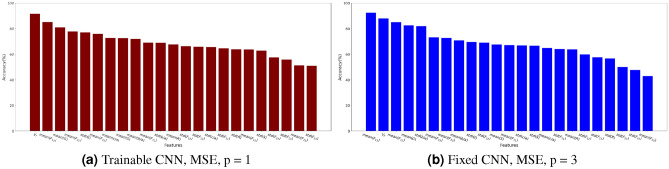
Accuracy of Model 1 with trainable and fixed convolutional layers. Results displayed by feature, for the two models that yield the highest mean accuracy across the features.

**Figure 14:**
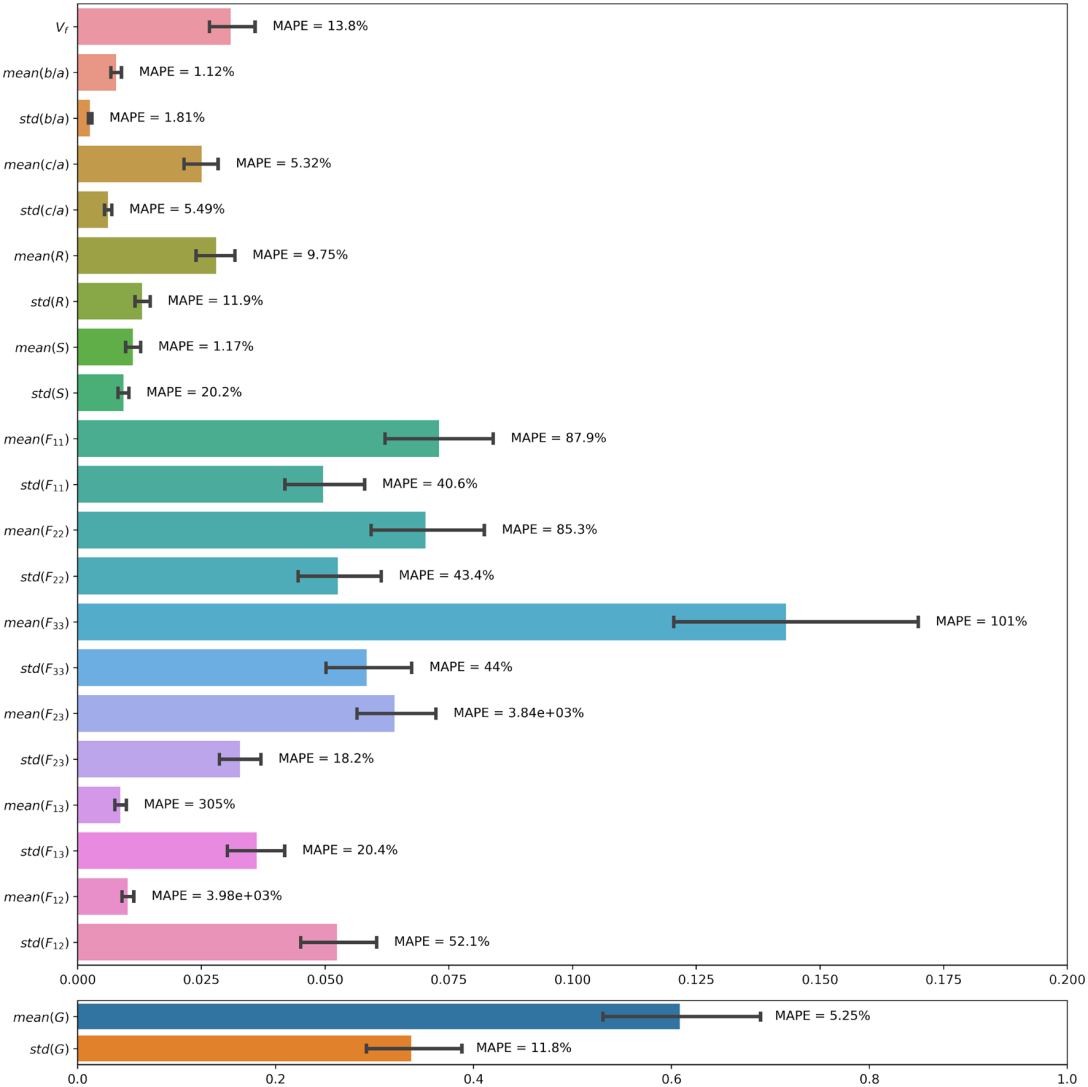
Model 1 performance to predict fabric descriptors from the test data set (MSE loss function, one 2D image per axis, trainable convolutional layers). Results are displayed in actual value (and not normalized value). The MAE is represented in colored bars, with its $95\%$ confidence interval. Each descriptor is also annotated with its MAPE.

Figure 13 provides the accuracy of the 23 estimated fabric descriptors for the two models that yield the highest mean accuracy: MSE loss with $p=1$ for trainable convolutional layers, and for MSE loss with $p=3$ for fixed convolutional layers. Features estimated with the highest and lowest accuracy are listed in Table 7 with the details of the models with which they were obtained. Figure 14 shows the MAE and MAPE of the features predicted from the testing data set, calculated by using Equation 11 for the model with highest mean ($68.65\%$). That model, which comprises trainable convolutional layers, uses the MSE loss function and takes 1 image per axis as input, yields a MAPE of 13.8% for the aggregate volume fraction, 5.25% for the mean aggregate size, less than 2% for the mean aspect ratios *a*/*b* and *c*/*a* and for the mean solidity, and 9.75% for the mean roundness. Most MAPEs for the standard deviations of those descriptors are in the range 5% - 20%. The means of the distributions of the components of the orientation matrix are predicted with a MAPE of the order of 100% or above, while the MAPEs of the standard deviations are mostly distributed between 20% and 50%. We conclude that Model 1 cannot be used to predict orientation, but gives satisfactory results to predict mean geometric descriptors. Standard deviations are harder to predict because their low values makes them similar to noise. The aggregate volume fraction is the lowest order descriptor, and yet, the MAPE associated to that descriptor is higher than that associated with higher order descriptors such as size, aspect ratio, roundness and solidity. The somewhat low performance of Model 1 for estimating the aggregate volume fraction may be due to the loss that is used, which gives equal importance to all descriptors. Highly correlated descriptors have a better chance to be well predicted, hence the higher performance for correlated geometric descriptors such as aspect ratio and roundness over aggregate volume fraction (see Fig. 4).

**Table 7:** Features which have highest/lowest accuracy with Model 1.

Descriptor	Estimator	Accuracy	Parameters
Features predicted with highest accuracy			
Global volume fraction	–	91.6%	Trainable CNN, MSE, p = 1
Grain size	Mean	82.5%	Fixed CNN, MSE, p=3
Orientation, $F_{13}$	Mean	92.3%	Fixed CNN, MSE, p = 3
Orientation, $F_{33}$	Mean	75.9%	Trainable CNN, MSE, p = 1
Orientation, $F_{22}$	Mean	77.7%	Trainable CNN, MSE, p = 1
Features predicted with lowest accuracy			
Orientation, $F_{13}$	Standard deviation	49.9%	Fixed CNN, MSE, p=3
Orientation, $F_{33}$	Standard deviation	47.63%	Fixed CNN, MSE, p=3
Orientation, $F_{23}$	Mean	42.9%	Fixed CNN, MSE, p=3

### Model 2: three channels of concatenated 2D images as input

#### Performance of the model trained and tested with 3 concatenated images per channel

**Figure 15:**
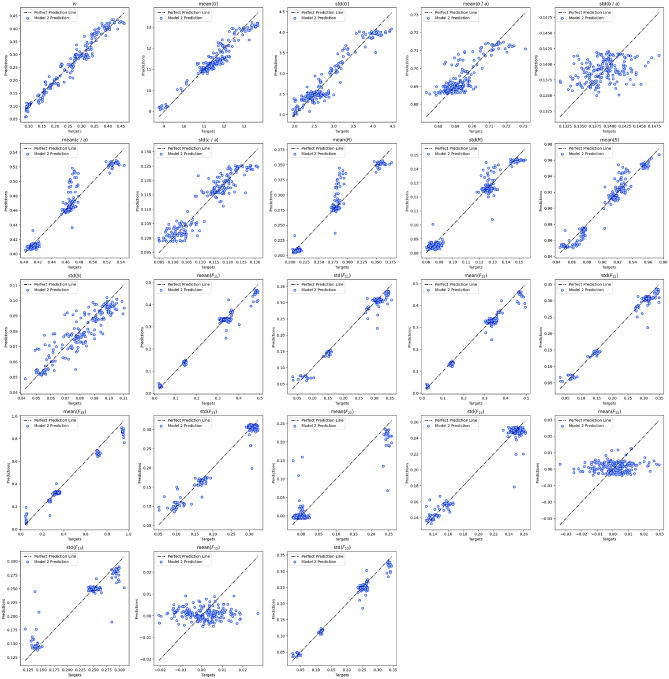
Microstructure descriptors predicted with Model 2 during testing versus ground truth ($p=3$).

**Figure 16:**
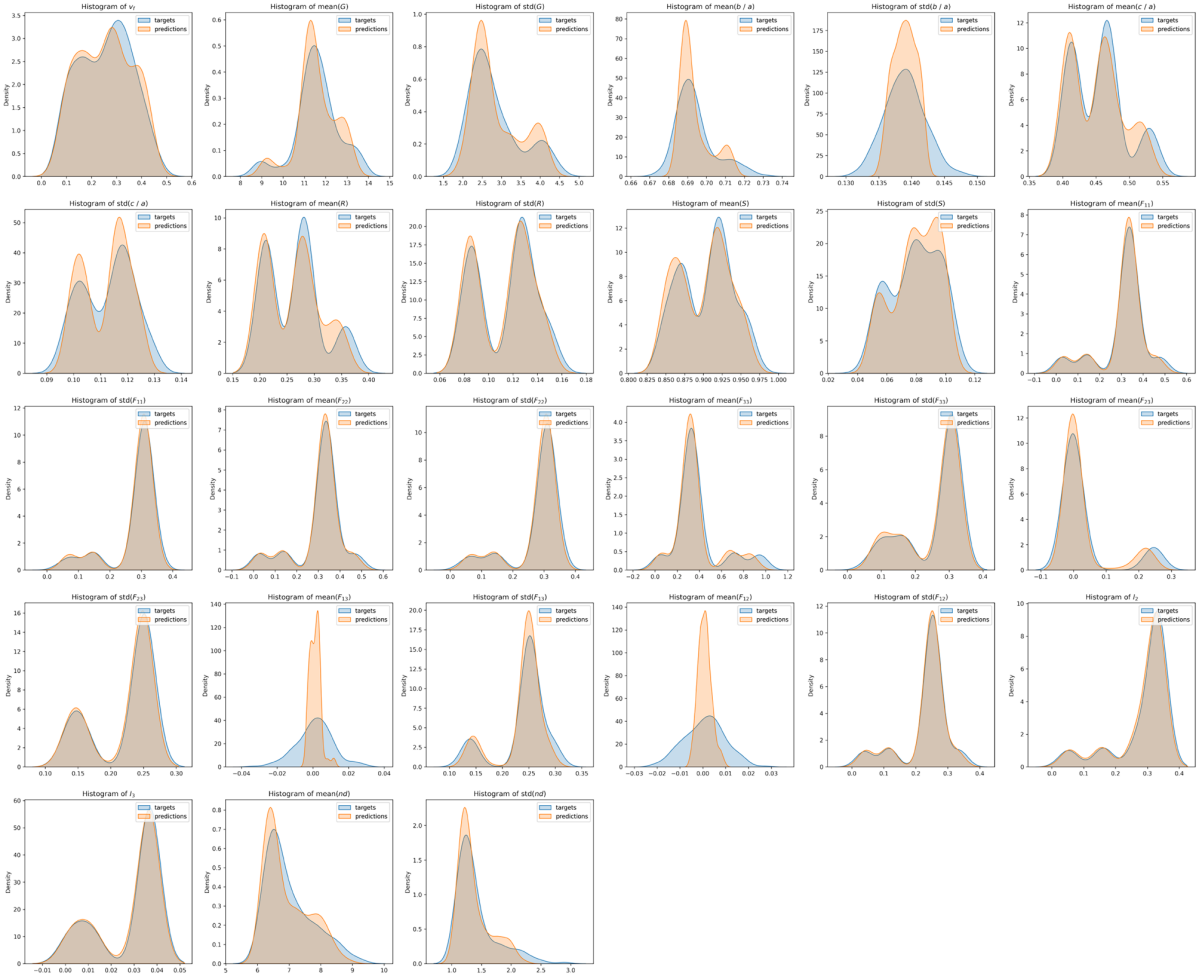
Distributions of the target and predicted descriptors in the test dataset after training Model 2 with inputs made of 3 concatenated images per channel. Microstructure descriptors are not normalized in these plots.

**Figure 17:**
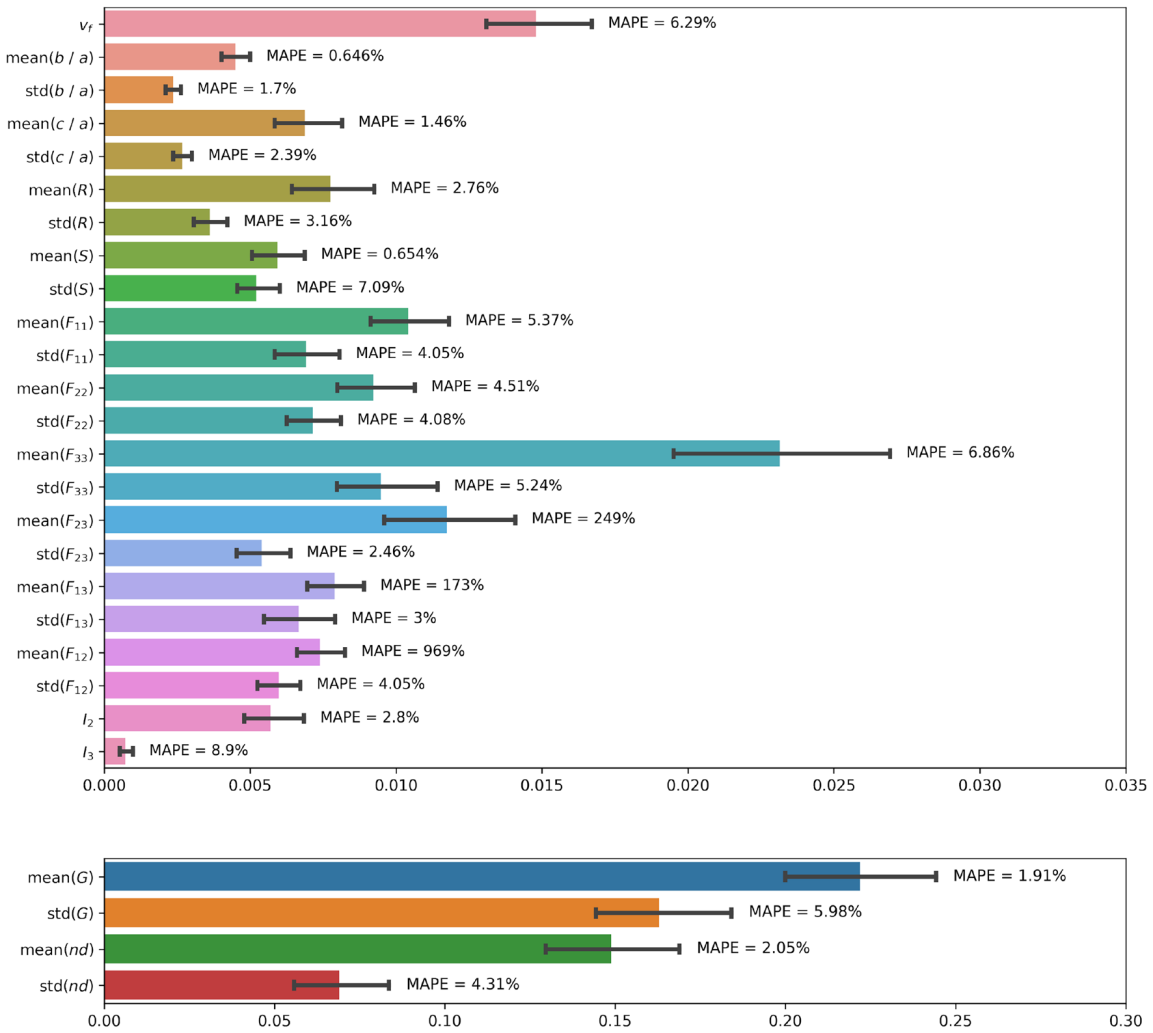
Model 2 performance to predict fabric descriptors from the test data set after training Model 2 with inputs made of 3 concatenated images per channel. Results are displayed in actual value (and not normalized value). The MAE is represented in colored bars, with its $95\%$ confidence interval. Each descriptor is also annotated with its MAPE.

Model 2 (based on VGG16) was first trained and tested only with inputs made of 3 concatenated images per channel. The MAE was 0.0276 on the test dataset. The value of each microstructure descriptor predicted with Model 2 during testing with three concatenated images per channel is plotted against its ground truth value in Fig. 15. Figure 16 shows the distributions of the target and predicted descriptors for the test dataset, in which the features have been converted to their actual values (instead of their normalized values) for a better physical understanding. The important overlap between the two suggests a high level of accuracy in the predictions. This high performance is confirmed by Fig. 17, which shows the prediction errors made for each of the 27 fabric descriptors under study. The prediction error made by the model oscillates between 1.5 and $7\%$ for most descriptors. In comparison, the prediction errors made by the mean algorithm are between 16 and $50\%$. Model 2 accurately estimates the means of descriptors like solidity (MAPE: $0.654\%$) and aggregate size (MAPE: $1.91\%$). But the model does not perform as well when estimating the average values of the off-diagonal coefficients of the orientation tensor (MAPE: $96.9\%$-$249\%$). We attribute this lower performance to the fact that the coefficients $F_{ij,\, i\ne j}$ have a low mean value and a low variance, which makes them difficult to estimate. We also notice that, except for the orientation tensor components, our model calculates means better than standard deviations in terms of MAPE. This is due to the fact that standard deviations of aggregate size, aspect ratio, roundness and solidity are often close to 0, resulting in higher MAPEs. Mathematically speaking, it is harder to predict second central moments (standard deviations) than first central moments (means). Overall, the invariants of the orientation tensor ($I_2$ and $I_3$, respectively) are predicted with excellent or good accuracy (MAPE of 2.8% and 8.9%, respectively), which suggests that the model can recognize geometric features regardless of the orientation of the input images.

#### Effect of the number of input images on the model performance

We retrained the model over 700 epochs with inputs made of 1 image per channel only, 5 images per channel, and 10 images per channel. The performance of Model 2 on the test datasets is summarized in Table 8. As expected, the MAE and MAPE decrease when the number of 2D images extracted in each direction of space increases. The increased performance comes with higher computational cost. For example, it takes about 13 hours to train the model with 10 images per channel as input. In average, the training time increases linearly with the number of images taken in each direction of space (*p*). The MAE decreases only marginally when *p* is increased beyond 3.

**Table 8:** Performance of Model 2 trained and tested with different input sizes.

Type of model	Time per epoch	MAE	MAPE
Mean algorithm	0	0.1863	30.1%
1 image per axis	7 s	0.0374	4.61%
3 image per axis	19 s	0.0276	3.59%
5 image per axis	41 s	0.0249	3.51%
10 image per axis	63 s	0.0227	3.38%

MAE averaged over the 27 fabric descriptors under study. MAPE averaged over 24 fabric descriptors: we excluded the off-diagonal coefficients of the orientation tensor, which have very low magnitude and low variance.

## Discussion

### On the prediction of orientation descriptors

Model 1 contains 2 to 28 more trainable parameters than Model 2 (depending on the number of slices used as input). Model 1 likely requires a larger dataset than Model 2 to be properly trained. Switching to Model 2 significantly improved the performance of the Visual Geometry Group (VGG) algorithm. Hence, the primary reason why orientation descriptors were not predicted with high accuracy with Model 1 was the lack of data available to train the deep neural network. Even with less parameters to train, Model 2 cannot predict the off-diagonal coefficients of the fabric tensor, for which the MAPE varies between 200% and 1,000%. The absolute mean values of $F_{11}$, $F_{22}$, $F_{33}$ are about 10 times higher than those of $F_{23}$, $F_{13}$ and $F_{12}$. Additionally, the off-the-diagonal coefficients of the fabric tensor exhibit low variance. Distributions with low mean values and low variance require a high-precision model to be accurately predicted, which may explain why even Model 2 is not achieving a good performance for $F_{23}$, $F_{13}$ and $F_{12}$. Model 2 performs well to predict the diagonal coefficients of the fabric tensor, since the MAPE for the means of $F_{11}$, $F_{22}$ and $F_{33}$ of is in the order of 4-6%. However, the MAPE obtained for the descriptors of shape, such as aspect ratio, roundness and sphericity, is 2-10 times lower. It may be possible to prune Model 2 further and achieve better performance. Another option is to train Model 2 several times to predict different sets of microstructure descriptors. For example, a set of VGG16 parameters could be used to predict shape descriptors only, and another set of VGG16 parameters could be used to predict orientation descriptors only. Besides the ratio number of parameters vs. number of predicted outputs, a possible explanation for the lower performance in predicting orientation descriptors is the choice of the loss function.

The Mean Squared Error (MSE) is widely used in vector regression for its simplicity, efficacy and versatility. But the MSE heavily penalizes larger errors, potentially leading to a model that is overfitted to the most common errors, at the expense of accurately predicting rarer or more complex cases. Experiments were conducted with the Mean Absolute Error (MAE), and generally yielded inferior outcomes. The Huber loss combines the benefits of the MSE and MAE, demonstrating reduced sensitivity to outliers compared to MSE. It could be interesting to finetune our models with this loss. The literature extensively investigates the impact of loss functions, particularly in regression contexts, thereby guiding studies to understand the effects of different losses^37^. For instance, the logCosh loss, akin to the Huber loss, often emerges as a viable compromise, though no loss function is guaranteed superior performance across all scenarios. Employing a custom loss function could enhance predictions of poorly predicted mechanical properties by focusing on specific data aspects. For example, for orientation descriptors, a loss function accounting for the cyclic nature of the orientation distribution could diminish prediction errors. Loss functions grounded in geometric distances or angular measures, such as the cosine loss, may more effectively capture the relationships between grain orientations in a biphasic structure. These approaches could offer an increased sensitivity to subtle orientation variations inadequately represented by the MSE.

Regardless, the second and third invariants of the fabric tensor are predicted with a MAPE of 2.8% and 8.9%, respectively, which indicates that despite a lower performance on orientation descriptors, Model 2 predicts well the relative orientation of microstructure features regardless of the orientation of the input image of the microstructure.

### On the interpretability of the models

The proposed models are biased towards highly correlated descriptors. This is because the VGG algorithms presented here are trained to minimize a loss function that is the sum of the errors made on all the fabric descriptors. All the errors in the sum are assigned the same weight, such that two highly correlated descriptors are twice more likely to be predicted accurately than a descriptor that is correlated to no other. According to Fig. 4, volume fraction and grain size are highly correlated. So are roundness, sphericity and the c/a aspect ratio. The diagonal coefficients of the fabric tensor and the fabric invariants are also highly correlated (positively or negatively). As expected, the least correlated descriptors, mainly, the off-diagonal coefficients of the fabric tensor, are predicted with less accuracy than the other descriptors. Descriptors that are correlated are predicted with similar accuracy. For example with Model 2, the range of MAPE is 0.5%-3% for all shape descriptors. However, the fact that some descriptors are highly correlated does not imply high model performance. For example, as noted earlier, the performance of Models 1 and 2 is lower for $F_{11}$, $F_{22}$, $F_{33}$, $I_2$ and $I_3$ than for shape descriptors. Mathematically, it may be beneficial to weigh the importance of the predicted microstructure features as a function of their correlations, to avoid bias. However, in practical applications, off-diagonal coefficients of the fabric tensor are less important than the combination of the fabric diagonal coefficients and invariants, which suffice to predict the orientation of features independently from the orientation of the input images. As a result, bias towards correlated descriptors that are interesting to the user may be an advantage.

### On the generalization performance of the models

Network pruning generally yields a good generalization performance for pre-trained networks like the VGG networks^38^ and allows reducing the number of parameters of the model. Most network pruning techniques consist in removing redundant neurons or connections^39–41^. For CNNs, network pruning either aims to remove redundant connections or to delete channels. Channel pruning reduces the feature map width, which may transform significantly the format of the input to the next layer, and make it challenging to achieve the desired accuracy. Additionally, the training time for any type of pruned network can be as high or higher than the original model^38^. Lastly, fine tuning pruned network remains a challenge because there is no theory to dictate which neuron or connection to prune. That is why knowledge interpretability is an active research area^38^. Network quantization is another network compression technique, in which the number of bits used to represent each weight is reduced^42,43^. Network quantization is known to yield good accuracy, except for very large CNNs^38^. Filters (also called structural matrices) can be applied to network layers to reduce the number of model parameters^44^. The transformations are similar to non-linear projections. In practice, structural matrices are difficult to find and may introduce bias in the model, hence lowering its performance^38^. Low-rank factorization is used to transform network layers into products of low-rank filters, which compresses the network and accelerates training and inference^45^. High dimensional DCT (discrete cosine transform) and wavelet systems using tensor products were successfully employed in deep learning. However, factorization requires extensive model retraining to achieve convergence. Additionally, this method relies on decomposition operations that have a high computational cost^38^. It is also possible to compress CNN models by applying transformations to a set of layers - a process called transferred convolutional filters. This method is known to achieve competitive performance for wide/flat architectures such as the VGG nets, but requires imposing prior human knowledge to the model, which may affect the stability of the models^38^. Lastly, parameter reduction can be achieved by knowledge distillation, which consists in training a deep learning model with thinner architecture to mimic the function learned by the original model with wide architecture^46^. The main drawback of knowledge distillation is that it is limited to networks with a softmax loss function. Additionally, the performance gain is lower than with other approaches such as pruning, quantization or factorization^38^.

Regardless of parameter reduction, generalization may be improved by training the deep learning model with smaller batches of data, because large-batch methods tend to converge to sharp minimizers of the training and testing functions^47^. Using mini-batches mobilizes more parallel computing resources, but improves convergence and accuracy^48^. It is also recommended to use an optimizer that partially adapts the learning rate as a function of a long history of the gradient of the loss function. In this study, the Adaptive momentum estimation method (Adam) was employed. Adam has demonstrated strong generalization capabilities. Once all the strategies above have been tested, generalization may be further enhanced by using the Partially adaptive momentum estimation method (Padam)^49^, which maintains a fast convergence rate, similar to the fully adaptive gradient method Adam, while achieving a generalization performance similar to that of the stochastic gradient descent (SGD) algorithm.

## Conclusions

In this study, we compared the performance of two custom neural networks from the Visual Geometry Group (VGG) to predict 3D fabric descriptors from a set of 2D images that represent slices of a 3D biphase microstructure in three orthogonal planes. The data set used for training and testing is a set of 600 3D microstructures of cemented aggregate assemblies that are created numerically. Each of these 600 3D microstructures is used to calculate a ground-truth vector of fabric descriptors such as the mean and standard deviation of aggregate size, aspect ratio and orientation. The input data are 2D images obtained by slicing the 3D microstructures in each direction of space at regular intervals.

The first model is a pruned version of VGG19 to which Axial Coronal Sagittal (ACS) convolutions are applied to allow formatting the input data as a single 3D image, i.e., as a concatenation of three objects. Each of those objects is the stack of 2D slices taken in the plane orthogonal to one direction of space, such that the input data set has a dimension $3\times p \times w \times h$, where *p* is the number of images taken in each of the planes of normal x, y and z, and *w* and *h* are the number of pixels of the 2D slices in the width and height directions. The custom ACS-VGG19 model contains fifteen convolutional layers organized in three blocks, and five fully connected layers. The second model is a customized version of the VGG16 model that contains 31 convolutional layers organized in three blocks, and three fully connected layers. The input to the VGG16 model is a stack of concatenated 2D images of dimensions $h \times (p \times w) \times 3$.

Overall, both models exhibit lower performance for orientation descriptors than for shape descriptors like aggregate size, aspect ratio, roundness and solidity. Both models perform better to predict the means than the standard deviations of shape descriptors. The ACS-VGG19 model that provides the best average accuracy across descriptors takes one image per direction as input, uses the MSE as a loss, and has fully trainable convolutional layers. The computational cost to train the custom ACS-VGG19 model increases linearly with *p* (the number of images extracted in each direction of space), and increasing *p* does not improve the performance of the model - or only does so marginally. For $p=1$, using the MSE loss and trainable convolutional layers, the custom ACS-VGG19 model provides a MAPE of 2 to 5% for the means of aggregate size, aspect ratios *a*/*b* and *c*/*a*, and solidity. Surprisingly, the lowest order descriptor, the aggregate volume fraction, is estimated with a MAPE of 13.8%, which is attributed to the bias given by the loss function towards highly-correlated descriptors. While the pruned ACS-VGG19 model cannot be used for estimating orientations, the custom VGG16 provides satisfactory estimates for all descriptors across the board, except for the off-diagonal components of the average orientation matrix, which exhibit low mean values and low variance and thus require a high-precision model to be accurately predicted. As an example, the custom VGG16 yields a MAPE of 2% or less for the means of aggregate size, distance to nearest neighbor, aspect ratios and solidity. The MAPE is less than 3% for the mean roundness, and in the range of 5-7% for the aggregate volume fraction and the mean diagonal components of the orientation matrix. It is interesting to note that the custom VGG16 model predicts the second and third invariants of the orientation matrix with a MAPE of 2.8% and 8.9%, respectively, which suggests that the model can predict orientation descriptors regardless of the orientation of the input images. The performance of Model 2 increases with the number of images taken as input in each direction of space. However, increasing *p* is h is not cost-effective beyond 3 images per axis because of the computational costs and the marginal loss reduction.

The custom VGG16 model (Model 2) performs better than the pruned version of ACS-VGG19 model (Model 1), likely because it contains less parameters than the ACS-VGG19 model. For example, for *p* = 1, Model 2 contains 16,048,729 parameters, compared to 52,561,751 parameters for Model 1. For *p* = 10, Model 2 contains 28 times less trainable parameters than Model 1. Model 1 contains heavy fully connected layers, which represent more than 98% of the trainable parameters. In comparison, the majority of the trainable parameters in Model 2 are found in the convolutional layers. Models with fewer parameters are able to converge better and faster, with less data. Intuitively, this is because finding the minimum of a loss function is easier when the loss depends on fewer parameters.

The independence of Model 2 to the orientation of the input impages implies that the proposed deep neural network could help optimize the number of slices acquired by computed tomography imaging for the characterization of anisotropic materials. The study reported in the manuscript indicates that using 3 slices per direction is optimal. The result may be extended to biphase materials with microstructures similar to those used in our dataset, mainly, cemented aggregates. However, for microstructures that exhibit very different geometric features, such as non-convex inclusions or fiber-like inclusions, it is necessary to re-train the models to optimize the number of input slices. The MAPE and the measure of accuracy proposed in the manuscript are useful tools to determine the optimum. We expect that the best trade-off between the quantity of input data (number of slices) and the number of microstructure descriptors predicted (model output) will partially depend on the quality of the input images. As a result, automating the selection of the number of input slices may not be feasible. A natural extension of this work is the adaptation of the proposed models for input data of variable format, which will allow training deep neural networks from inputs that contain various numbers of images per direction, different numbers of images in different directions, and unequally spaced slices.

## Data Availability

Accession codes: https://github.com/MatiasEtcheve/microstructure-reconstruction. Accession dataset: https://github.com/MatiasEtcheve/microstructure-reconstruction/tree/master/REV1_600.
